# A Placebo-Controlled, Pseudo-Randomized, Crossover Trial of Botanical Agents for Gulf War Illness: Resveratrol (*Polygonum cuspidatum*), Luteolin, and Fisetin (*Rhus succedanea*)

**DOI:** 10.3390/ijerph18052483

**Published:** 2021-03-03

**Authors:** Kathleen S. Hodgin, Emily K. Donovan, Sophia Kekes-Szabo, Joanne C. Lin, Joseph Feick, Rebecca L. Massey, Timothy J. Ness, Jarred W. Younger

**Affiliations:** 1 Department of Psychology, University of Alabama at Birmingham, CH 233, 1300 University Boulevard, Birmingham, AL 35233, USA; kathleenhodgin@uabmc.edu; 2 Department of Psychology, Virginia Commonwealth University, White House, 806 West Franklin Street, Richmond, VA 23284, USA; donovanek@mymail.vcu.edu; 3 Department of Psychology, Vanderbilt University, PMB 407817, 2301 Vanderbilt Place, Nashville, TN 37240-7817, USA; sophia.kekes-szabo@vanderbilt.edu; 4 School of Pharmacy, University of Auckland, 85 Park Road, Grafton, Auckland 1023, New Zealand; joanne.lin@auckland.ac.nz; 5 Double Oak Mountain Pharmacy, 5510 Highway 280, Suite 123, Birmingham, AL 35242, USA; domprx@gmail.com; 6 UAB School of Medicine, University of Alabama at Birmingham, 1670 University Boulevard, Birmingham, AL 35223, USA; rlmassey@uab.edu; 7 Department of Anesthesiology and Perioperative Medicine, University of Alabama at Birmingham, BMR2-208, 901 19th Street South, Birmingham, AL 35205, USA; tness@uabmc.edu

**Keywords:** resveratrol, luteolin, fisetin

## Abstract

A chronic multi-symptom illness of unknown etiology, Gulf War Illness (GWI) affects 175,000 to 250,000 veterans of the Gulf War. Because inflammation has suspected involvement in the pathophysiology of GWI, botanical treatments that target inflammation may be beneficial in reducing symptoms. No FDA-approved treatments currently exist for GWI, and rapid prioritization of agents for future efficacy testing is important. This study is part of a larger project that screened nine different botanical compounds with purported anti-inflammatory properties for potential treatment of GWI. We tested three botanicals (resveratrol [*Polygonum cuspidatum*], luteolin, and fisetin [*Rhus succedanea*]) on symptom severity of GWI in this placebo-controlled, pseudo-randomized clinical trial. Twenty-one male veterans with GWI completed the study protocol, which consisted of 1 month (30 days ± 3) of baseline symptom reports, 1 month of placebo, 1 month of lower-dose botanical, and 1 month of higher-dose botanical. Participants completed up to 3 different botanicals, repeating the placebo, lower-dose, and higher-dose cycle for each botanical assigned. Linear mixed models were used for analyses. Resveratrol reduced GWI symptom severity significantly more than placebo at both the lower (*p* = 0.035) and higher (*p* = 0.004) dosages. Luteolin did not decrease symptom severity more than placebo at either the lower (*p* = 0.718) or higher dosages (*p* = 0.492). Similarly, fisetin did not reduce symptom severity at either the lower (*p* = 0.504) or higher (*p* = 0.616) dosages. Preliminary findings from this screening study suggest that resveratrol may be beneficial in reducing symptoms of GWI and should be prioritized for future testing. Larger trials are required to determine efficacy, response rates, durability of effects, safety, and optimal dosage. This trial was registered on ClinicalTrials.gov (NCT02909686) on 13 September 2016.

## 1. Introduction

With a lack of standard treatment recommendations [1], Gulf War Illness (GWI) continues to affect about a third of the 700,000 veterans deployed to the 1990–91 Persian Gulf War [2]. These veterans reported fatigue, pain, cognitive dysfunction, gastrointestinal issues, respiratory problems, and skin abnormalities during or shortly after deployment [3].

Multiple studies implicate neuroimmune dysregulation as a driver of GWI symptoms [4,5,6,7], but the precise etiology and pathophysiological mechanisms remain unknown. In an animal model of GWI, proinflammatory cytokines and high mobility group box 1 (HMGB) protein are increased in both the brain and circulating neuron-derived extracellular vesicles [8]. In veterans with GWI, elevated levels of both brain-derived neurotrophic factor (BDNF) and C-reactive protein (CRP) have been reported [9]. We have also observed increased interleukin (IL)-1β and IL-15 among veterans with GWI on days with higher fatigue [10]. Further, increased uptake of translocator protein (TSPO), a marker of microglial activation, has been found in the brains of veterans with GWI in a positron emission tomography study using [^11^C] PBR28 [11].

Botanical supplements with known anti-inflammatory properties may have value in treating GWI, but no study has been conducted to assess which compounds ought to be prioritized for efficacy testing. In this pseudo-randomized, placebo-controlled, human in vivo study, we aimed to rapidly screen botanical agents as possible GWI treatment candidates. This report is part of a larger study investigating several agents with suspected anti-inflammatory properties: curcumin, boswellia, French maritime pine bark, epimedium, fisetin, luteolin, reishi mushroom, resveratrol, and stinging nettle. In this report, the effects of the flavonoids/phenol compounds (resveratrol [*Polygonum cuspidatum*], luteolin, and fisetin [*Rhus succedanea*]) are discussed. Data from the other six botanicals studied are presented in two separate reports based on other shared properties of the compounds. The decision to present study results in this manner was made prior to any analyses performed.

Resveratrol (3,4′,5-trihydroxystilbene) is a dietary polyphenol found in the skin of red grapes, berries, peanuts, and other foods. Preclinical research has revealed neuroprotective [12], anti-inflammatory [13], and antioxidant [14] effects of resveratrol. In lipopolysaccharide (LPS)-induced neuroinflammation, resveratrol has been shown to protect cortical neurons by inhibiting microglial activation and associated production of pro-inflammatory and cytotoxic factors such as tumor necrosis factor alpha (TNF-α), nitric oxide, and IL-1β [13]. In an animal model of age-related cognitive changes, resveratrol treatment was associated with improved cognitive function accompanied by enhanced neurogenesis and microvasculature, reduced microglial activation, and decreased astrocyte hypertrophy in the hippocampus [15]. A meta-analysis of randomized controlled trials of resveratrol reported significant reductions in high sensitivity CRP (hsCRP) and TNF-α after resveratrol treatment in both healthy adults and adults with chronic medical conditions (e.g., cardiovascular disease, coronary artery disease, chronic obstructive pulmonary disease) [16]. Resveratrol is also associated with improvements in memory in older adults [17] and in overall cognitive performance [18] in post-menopausal women.

Luteolin (3′,4′,5,7-tetrahydroxyflavone) is a flavonoid found in many plants including parsley, oregano, celery, and peppermint [19]. Luteolin acts as an anti-inflammatory and antioxidant agent with the ability to cross the blood–brain barrier [20]. It has potent inhibitory effects on microglia [21] and has been shown to reduce IL-6 production [22], inhibit LPS-induced nitric oxide expression [23], and reduce CD40 expression on microglia [24]. Luteolin has also been shown to decrease pro-inflammatory cytokines such as IL-1β, TNF-α, and IL-17, and increase the anti-inflammatory cytokine IL-10 [25]. These anti-inflammatory effects are likely mediated in part via inhibition of nuclear factor kappa light chain enhancer of activated B cells (NF-κΒ) signaling [25].

Fisetin (3,3′,4′,7-tetrahydroxyflavone), another flavonoid, is most highly concentrated in strawberries and is also found in apples, persimmons, and onions [26]. Like luteolin and resveratrol, fisetin has purported anti-inflammatory [27], antioxidant [28], and neuroprotective properties [29]. Fisetin exerts anti-inflammatory effects on mast cells [30] and suppresses activation of microglia when stimulated by LPS, blocking LPS-induced production of nitric oxide, TNF-α and prostaglandin E2 [31]. Fisetin’s anti-inflammatory properties have been shown to protect against aluminum chloride (AlCl3)–induced neurotoxicity in humans [32].

In this study, we aimed to screen anti-inflammatory botanical agents for future efficacy testing for GWI. Resveratrol, luteolin, and fisetin were each tested in lower-dose and higher-dose conditions against baseline and placebo periods. Our primary outcome measure was self-reported overall GWI symptom severity. We hypothesized that the overall GWI symptom severity would be significantly reduced during the lower- and higher-dose botanical conditions in comparison to baseline and placebo. We also examined the secondary outcomes of self-reported pain and fatigue severity.

## 2. Materials and Methods

The three agents discussed in this report were part of a larger program of study that investigated the effects of nine anti-inflammatory botanicals on GWI symptoms. The study protocol was approved by the University of Alabama at Birmingham (UAB) Institutional Review Board on 30 June 2015 (F150318011), and this study was registered on ClinicalTrials.gov on 21 September 2016 (NCT02909686). Individuals were recruited via radio, print, and online advertisements. All participants provided written informed consent. Participants were randomized to receive up to three botanical compounds in total over the course of the study, such that each botanical was trialed by at least ten individuals. Participants recorded symptoms daily throughout the baseline, placebo, and treatment periods.

### 2.1. Participants

Inclusion criteria included the following: male sex; age 37–65; presence in the Persian Gulf region between August 1990 and August 1991; ability to come to the study site for 11 monthly visits; ability to receive a venous blood draw; and successful completion of ≥80% of daily symptom reports over the baseline period. All participants had to fulfill Kansas Gulf War Illness case criteria [33], with an exception for well-controlled diabetes mellitus type 2 (A1C ≤ 9%) and in one case for an individual with a history of cancer outside of the last 5 years (Hodgkin’s lymphoma in remission for 20 years). In such cases of unclear decisions for inclusion, the author of the Kansas GWI case criteria was consulted for guidance.

Individuals were excluded from study participation for the following: current daily use of opioid or anti-inflammatory medications; use of nitroglycerine or lithium; history of anaphylaxis to any botanical used in the study; hypotension (<90/60 mmHg) or history of cardiovascular disease; diagnosis of rheumatologic or autoimmune disease; blood or clotting disorder; current litigation of worker’s compensation claim; and inability to read and understand English. Baseline exclusionary criteria also included acute infection (body temperature above 100.4 °F), positive rheumatoid factor (RF), positive antinuclear antibody (ANA), erythrocyte sedimentation rate (ESR) > 40 mm/hr, and CRP > 10.0 mg/L. Individuals with current severe depressive symptoms as suggested by a depression subscale score ≥ 16 on the Hospital Anxiety and Depression Scale (HADS; [34]) were excluded. Current Posttraumatic Stress Disorder (PTSD) was also exclusionary. Individuals with scores ≥ 50 on the PTSD Checklist–Military Version (PCL-M; [35]) were assessed for the presence of current PTSD with the Clinician Administered PTSD Scale (CAPS-5; [36]).

### 2.2. Treatments

Resveratrol was sourced from Pure Encapsulations (Sudbury, MA, USA) and was standardized to contain 20% trans resveratrol. Luteolin was obtained from A.P.I. Solutions (Daphne, AL, USA), and fisetin was sourced from VitaCost (Boca Raton, FL, USA). All botanicals were sent to Double Oak Mountain Pharmacy in Birmingham, AL, USA, where they were re-encapsulated in size 0 or 00 opaque blue gelatin capsules. Microcrystalline cellulose was used as filler for the placebo treatment, with the same blue covering capsules as the botanical compounds, so that all treatments appeared identical. All capsules were placed in standardized QUBE Weekly (28 cavity) Cold Seal Compliance Blister Packs (Pharmacy Automation Supplies, Romeoville, IL, USA).

Treatments were administered twice a day. The lower dose for resveratrol was 200 mg/day (200 mg in morning and 0 mg in evening), and the higher dose for resveratrol was 600 mg/day (400 mg in morning; 200 mg in evening). For luteolin, the lower dose was 200 mg/day, and the higher dose was 400 mg/day. Fisetin was administered at 200 mg/day for the lower dose and 800 mg/day for the higher dose. For both luteolin and fisetin, total dosage was evenly split between morning and night doses.

### 2.3. Study Protocol

**Figure 1:**
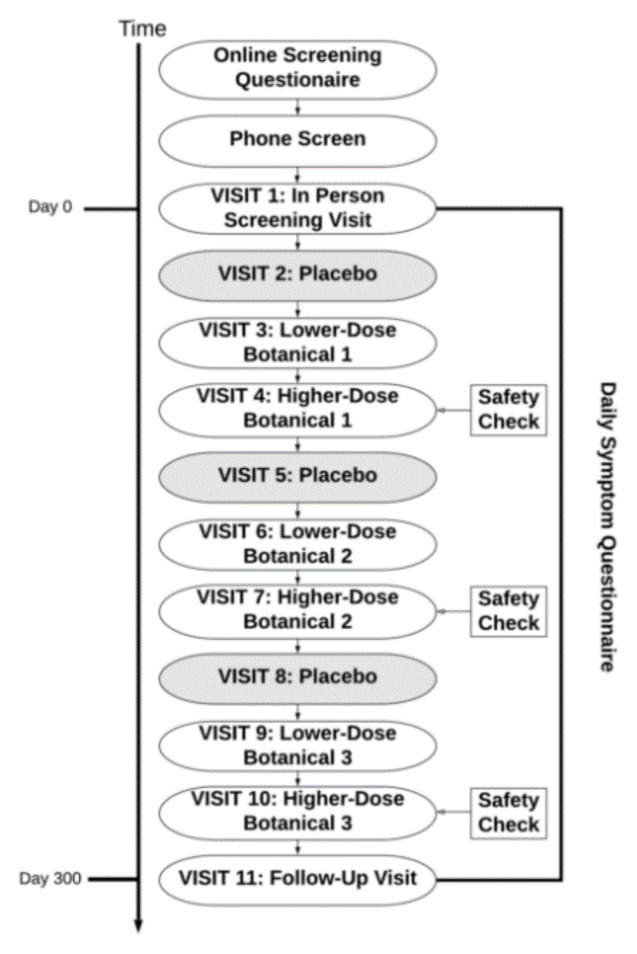
Study protocol. Each participant completed testing of up to three botanicals. For each botanical, there was a placebo condition, followed by lower-dose botanical and higher-dose botanical conditions. The period of time between visits was 30 ± 3 days. Some participants re-enrolled in the study after completion, receiving up to a maximum of six botanicals.

The study protocol is depicted in Figure 1. Interested individuals completed an online pre-screening questionnaire prior to a phone interview with research personnel in order to assess for initial eligibility. For individuals who met initial inclusion criteria, an in-person screening visit was held at the UAB Center for Clinical and Translational Science Clinical Research Unit (CRU). All participants provided written informed consent at the in-person screening. Baseline study questionnaires were administered on a tablet device, and vital signs and venous blood samples were collected by CRU staff to assess for exclusionary laboratory values. Participants were also provided with computer tablet devices to take home with them in order to complete once daily symptom reports during the entire study period. A one-month baseline symptom reporting period began immediately following the in-person screening visit. Participants were required to complete at least 80% of daily symptom reports during this period to remain eligible for the study.

Pseudo-randomization was then performed so that participants were assigned to receive up to three out of the nine botanical compounds, as shown in Figure 1. Pseudo-randomization was done in such a way to prevent drug interactions that contraindicated the use of the botanical in a given participant. There were no contraindications for the use of luteolin. Because blood glucose may be lowered by resveratrol [37] and fisetin [38], individuals taking medications for diabetes, or with evidence of prediabetes or diabetes, were excluded from taking these agents. Additionally, individuals with clotting disorders, hypotension, or those taking anticoagulants or antihypertensive medications were excluded from taking resveratrol, due to its effects on blood pressure [39,40]. Prophylactic use of 81 mg aspirin daily was permitted.

All study visits were held at the CRU and were conducted once a month for 10 months after completion of the baseline period. Participants received kits at each visit containing a one-month supply of either placebo or botanical capsules. Kidney and liver function were monitored with blood draws conducted at visits 4, 7, and 10. The following laboratory values were tested at those visits: sodium, potassium, chloride, bicarbonate, anion gap, glucose, blood urea nitrogen, creatinine, calcium, phosphorus, albumin, total protein, total bilirubin, direct bilirubin, indirect bilirubin, alkaline phosphatase, aspartate aminotransferase (AST), and alanine aminotransferase (ALT).

The same protocol (one month of placebo, one month of lower-dose botanical, and one month of higher-dose botanical) was completed for each botanical. For safety reasons, lower-dose conditions always preceded higher-dose conditions, allowing adverse effects to be detected before a higher dose of the agent was used. Only the study pharmacist was unblinded to the assigned botanicals. All other study staff were aware of the design condition order, but were blinded to the specific botanical taken by each participant. Participants were blinded to both the administration order (placebo, lower-dose, and higher-dose) and the botanical compounds.

Treatment adherence was monitored at each study visit. Each month, participants were instructed to return their blister packs. Study staff checked for any missed doses and discussed with participants the importance of adhering to the administration regimen. Participants were provided with the option of re-enrolling in the study after completing the protocol, such that they could be assigned up to a total of six out of the nine compounds.

### 2.4. Screening Measures

Participants were screened for GWI using the Kansas Gulf War Illness (GWI) case definition [33], in which symptoms are rated in severity from 0 (none) to 3 (severe). Inclusion criteria were met if an individual obtained a score of 2 or greater (indicating at least one moderate symptom or two mild symptoms) across three or more of six symptom domains (pain, fatigue, neurological/cognitive/mood, gastrointestinal, respiratory, and skin). Symptoms were required to have begun during or after Gulf War service. Chronic medical conditions not associated with Gulf War military service (including heart disease, stroke, lupus, multiple sclerosis, cancer [other than skin cancer], melanoma, and liver disease) were exclusionary for case definition. Due to the possibility of impacting a participant’s ability to report symptoms, the following other conditions were exclusionary per case criteria: bipolar or manic depression, schizophrenia, and recent hospitalization for alcohol or drug dependence, depression, or PTSD.

Participants were screened for symptoms of severe depression with the HADS [34]. This questionnaire was part of the online pre-screening process and was completed using the Qualtrics Research Suite Online Application. The 14 items of the HADS contain subscales for Anxiety (HADS-A) and Depression (HADS-D), each with 7 items. Items are rated from 0 to 3, and reverse scoring is used for 5 of 14 total items. Higher ratings suggest greater symptomatology. All ratings are summed for a total score ranging from 0 to 42.

Participants were also screened for severe symptoms of PTSD using the PCL-M [35]. Like the HADS, the PCL-M was completed during the online pre-screening process using the Qualtrics Research Suite Application. The 17-item PCL-M reflects PTSD diagnostic criteria of the fourth edition of the Diagnostic and Statistical Manual of Mental Disorders (DSM-IV; [41]). Each item inquires about trauma and stress symptoms associated with military experiences (e.g., Repeated, disturbing memories, thoughts, or images of a stressful military experience?) and is rated on a Likert scale from 1 (not at all) to 5 (extremely). Scores of 50 or greater suggest an increased likelihood of meeting DSM-IV diagnostic criteria for PTSD [42].

If a PCL-M score of 50 or above was obtained, potential participants were evaluated further for current PTSD symptoms using the last month version of the CAPS-5 [36]. Personnel of the UAB Office of Psychiatric Clinical Research administered the CAPS-5 at the CRU during the in-person screening visit. The CAPS-5 is a structured interview consisting of 30 items assessing PTSD symptom severity and diagnostic status (symptom duration and onset, distress levels, impact on social and occupational functioning, response validity, and dissociative subtype symptoms). Items are rated with a severity score based on frequency and intensity of symptoms. A symptom that meets the threshold for diagnosis of current PTSD is suggested by a severity rating of 2 (“Moderate/threshold”).

### 2.5. Main Outcome Measures

Participants reported their symptoms once daily in the evening via the Qualtrics Research Suite Offline Application (Qualtrics, Provo, UT, USA). The reports consisted of visual analog scale (VAS) ratings of symptoms scored from 0 to 100. Given that GWI consists of multiple idiosyncratic symptoms, a single-item severity score was chosen as the primary outcome to provide a common measure for GWI symptom severity. The main outcome was assessed with the following question, “Overall, how severe have your symptoms been today?” with “Not severe at all” fixed on the far left and “Extremely severe” fixed on the far right. Research personnel instructed participants to rate their daily overall GWI symptom severity based on their particular GWI symptoms, encompassing all possible six GWI domains.

### 2.6. Secondary Outcome Measures

In addition to the GWI symptom severity item, participants responded to several other daily single-item measures. These items assessed specific symptoms including pain, fatigue, cognitive dysfunction, depressed mood, skin problems, respiratory complaints, and gastrointestinal issues. A sufficient number of participants endorsed the pain and fatigue items so that statistical analyses could be applied. Participants responded to pain and fatigue questions using two separate 0–100 VAS ratings. To assess pain, participants were asked, “Overall, how severe is your pain?”, with “No pain at all” fixed on the left and “Severe pain” on the right. The item regarding fatigue asked, “How fatigued have you felt today?”, with “Not fatigued at all” fixed on the left and “Severely fatigued” on the right.

### 2.7. Statistical Analyses

SPSS Statistics for Windows, version 24 (IBM Corp., Armonk, NY, USA) was used to conduct analyses. Linear mixed models (LMM) were utilized to test differences in self-reported symptom severity between the four conditions. A separate model was tested for each of the three botanicals. Subject ID was entered as the subject identifier, with all individual longitudinal data nested within-person. The participant’s day in the study was entered as the index variable for repeated measures. Compound symmetry was selected as the repeated measures covariance type. The AR (1) autoregressive covariance type was also considered, but it did not improve the Akaike information criterion (AIC) or Bayesian information criterion (BIC) for model performance. The dependent variable was daily GWI symptom severity (0–100). Treatment condition (baseline, placebo, lower-dose, and higher-dose) was entered as a fixed factor. The last 14 days of each condition were selected for analyses to permit time for clinical effects of the treatments to occur. A restricted maximum likelihood (REML) estimation approach was used. Statistical significance was set at *p* < 0.05 for all tests. The secondary analyses were conducted using the same approach.

We performed a-priori power analyses using G*Power 3 [43] to estimate sample size. Each of the nine botanical trials was powered to detect a medium effect (Cohen’s d = 0.5) at 0.99 power with *p* = 0.05 threshold for significance. Given 56 repeated outcome measurements per participant (with a repeated measures correlation of 0.5), each trial required 10 individuals to obtain 0.99 power. Our studies were not powered (0.42 predicted power) to detect small effects (Cohen’s d = 0.25).

## 3. Results

### 3.1. Participants

**Figure 2:**
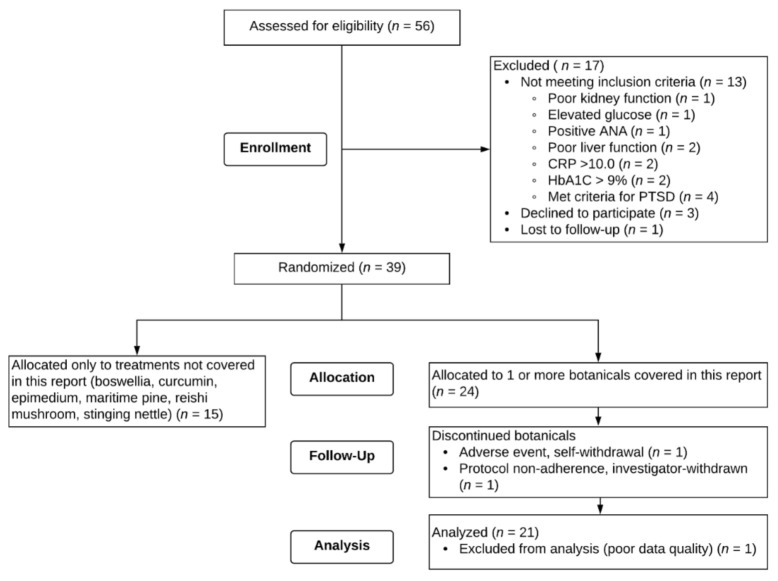
CONSORT Flow diagram. Twenty-four individuals were randomized to at least one of the treatments covered in this report.

Fifty-six male veterans provided written consent to participate. A flow chart of recruitment, enrollment, and attrition for the study is shown in Figure 2. Of the 56 individuals who consented to the study, 13 were excluded due to screening criteria, 1 was lost to follow-up after the screening visit, and 3 self-withdrew before beginning any treatment. Thirty-nine participants remained eligible and were randomized to receive any of the 9 botanicals tested. Fifteen veterans were randomized to receive only botanicals not included in this report. Twenty-four veterans were assigned to receive at least one of the three botanicals discussed herein (resveratrol, luteolin, and fisetin).

Of the 24 participants randomized to receive resveratrol, luteolin, and/or fisetin, 1 participant self-withdrew from the study. Shortly after initiating fisetin, the participant developed an adverse event (swollen lymph node due to bacterial infection), discontinued the capsules, and self-withdrew from the study. This adverse event was determined to not be related to the study treatment. One participant who took luteolin was investigator-withdrawn for failure to adhere to study protocol. One participant who took resveratrol and luteolin and completed the study was excluded from all analyses due to poor data quality (i.e., retrospective reporting).

A total of twenty-one male veterans, aged 46 to 57 (M = 49.76, SD = 2.76), were included in analyses. Participants were randomly assigned to receive three out of the nine total botanicals and as such are also represented in the other reports, depending on which botanicals they were assigned. Of the twenty-one participants included in analyses for this report, two participants were assigned to all three botanicals discussed here (resveratrol, luteolin, and fisetin). Seven of the twenty-one participants were assigned to two of the three botanicals, and twelve of the twenty-one participants were assigned to only one of the botanicals in this report. Nineteen (90.5%) of the participants identified as White, non-Hispanic, and two (9.5%) identified as Black, non-Hispanic. All participants (apart from the participant who withdrew after initiating fisetin, as noted above) completed at least the baseline period and an entire course of capsules for a botanical (placebo, lower-dose, and higher-dose). One participant completed the baseline, placebo, and lower-dose periods for fisetin, but discontinued the higher-dose fisetin after taking one week of capsules due to development of worsening nausea and gastroesophageal reflux disease (GERD). This event did not result in study withdrawal and resolved within a week of discontinuing capsules. Only data from the baseline, placebo, and lower-dose fisetin periods for this participant were included in analyses. One participant (who took resveratrol, luteolin, and fisetin) reported all zeros for GWI symptom severity over the entire study duration and thus could not be included in analyses for the main outcome question.

### 3.2. Resveratrol

**Figure 3:**
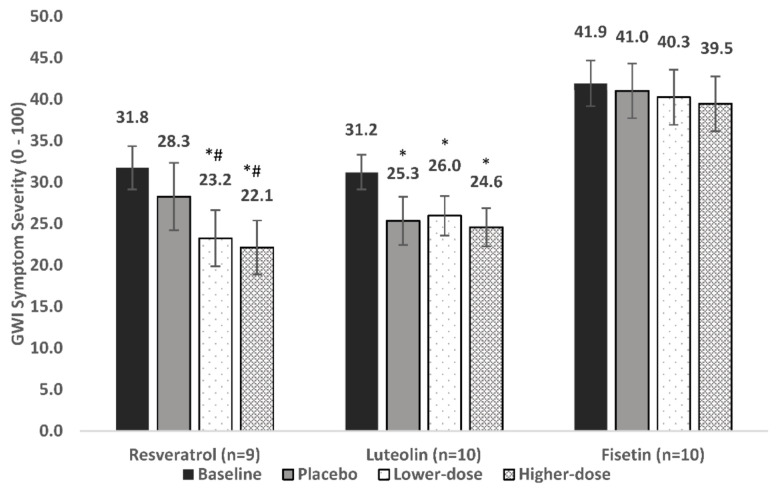
Main treatment effects of resveratrol, luteolin, and fisetin on Gulf War Illness (GWI) symptom severity. Average symptom levels (0–100) are presented for the baseline, placebo, lower-dose, and higher-dose conditions. * Significantly lower than baseline (*p* < 0.05); *^#^ significantly lower than baseline and placebo (*p* < 0.05).

For the nine individuals included in analyses, baseline overall symptom severity was 31.8. Symptom severity was 28.3 during placebo, 23.2 in lower-dose resveratrol, and 22.1 in higher-dose resveratrol, representing an 11%, 26.8%, and 30.3% decrease in severity, respectively (Figure 3).

The LMM showed a significant main effect for condition [F (3, 592) = 6.9, *p* = 0.0002]. Post-hoc contrasts showed that placebo did not reduce symptoms significantly from baseline (*p* = 0.606). Lower-dose resveratrol reduced symptom severity from both baseline (*p* = 0.003) and placebo (*p* = 0.035). Higher-dose resveratrol also reduced symptom severity from baseline (*p* < 0.0001) and placebo (*p* = 0.004). There was no significant difference between lower-dose and higher-dose resveratrol (*p* = 0.444).

### 3.3. Luteolin

Ten individuals were included in analyses of the primary outcome. Baseline symptom severity was 31.2. Symptom severity was 25.3 during placebo, 26.0 during lower-dose luteolin, and 24.6 during higher-dose luteolin, representing an 18.8%, 16.8%, and 21.3% decrease in severity, respectively (Figure 3).

The LMM showed a significant main effect for condition [F (3, 624) = 11.7, *p* < 0.0001]. Post-hoc contrasts showed that all conditions lowered symptom severity from baseline: placebo (*p* = 0.0001), lower-dose luteolin (*p* < 0.0001), and higher-dose luteolin (*p* < 0.0001); however, neither lower-dose luteolin (*p* = 0.718) nor higher-dose luteolin (*p* = 0.492) was significantly better than placebo.

### 3.4. Fisetin

Ten individuals were included in analyses of the primary outcome. Baseline symptom severity was 41.9. Symptom severity was 41.0 during placebo, 40.3 during lower-dose fisetin, and 39.5 during higher-dose fisetin, representing a 2.1%, 2.1%, and 5.9% decrease in severity, respectively (Figure 3).

The LMM did not show a significant main effect for condition [F (3, 559) = 0.175, *p* = 0.913). Placebo, lower-dose fisetin, and higher-dose fisetin were not associated with a significant decrease of symptoms from baseline (*p* = 0.794, 0.504, and 0.616, respectively).

### 3.5. Secondary Outcomes

Effects of the three botanicals on pain and fatigue severity are presented in Table 1. Both lower-dose and higher-dose resveratrol reduced pain significantly more than placebo, but neither reduced fatigue over placebo. Luteolin had no significant impact over placebo for pain or fatigue. Fisetin likewise had no significant effect on pain or fatigue over placebo.

### 3.6. Adverse Events

Fourteen of the 21 total participants reported adverse events (AEs) that could be possibly attributed to the treatment. AE severity was mild to moderate. Worsening GERD was the most commonly reported side effect. Migraine was endorsed by two participants during the placebo condition, and worsening fatigue was reported by two participants while taking the higher dose of fisetin. No other side effects were endorsed by more than one participant for each condition. Table 2 provides a summary of adverse events.

## 4. Discussion

We examined the effects of resveratrol, luteolin, and fisetin on symptoms of GWI. No significant adverse effects were found, and all three botanicals were well tolerated. Results suggest that both lower- and higher-dose resveratrol had significant effects on overall GWI symptom severity, as well as pain. Neither luteolin nor fisetin at lower or higher doses demonstrated significant effects on symptoms of GWI.

We utilized a unique study design for this clinical trial, screening a total of nine different botanical compounds over a two-year period. Our intention was to rapidly identify treatments with high potential for GWI symptom reduction, aiding in their prioritization for full efficacy study. Sample sizes were small. Thus, study findings are preliminary only and cannot form the basis for treatment recommendations.

We are not aware of any previously published clinical trials investigating resveratrol for GWI or potentially related chronic, multi-symptom illnesses such as fibromyalgia (FM) and myalgic encephalomyelitis/chronic fatigue syndrome (ME/CFS). One clinical trial of concord grape juice consumption has been conducted among veterans with GWI; findings suggested potential benefit in one measure of executive function but no differences in fatigue or other outcomes [44]. However, the 16 ounces of grape juice administered daily in that study contained approximately 0.16 mg of resveratrol [45], whereas participants in the current trial received 200 mg/day for one month followed by another month of 600 mg/day. Prior studies with resveratrol have been conducted in other pain conditions, such as knee osteoarthritis (OA). In a study among knee OA patients, resveratrol as an adjunct treatment to meloxicam significantly reduced pain severity when compared to meloxicam with placebo [46]. Inflammatory markers (TNF-α, IL-1β, IL-6, and hsCRP) also significantly declined in the group treated with resveratrol [46]. We found that resveratrol, at both higher and lower doses, had significant effects on self-reported pain, as well as overall symptom severity in veterans with GWI. It is possible that these effects of resveratrol occur via anti-inflammatory mechanisms, such as decreased glial activation and inhibition of cyclooxygenase [47]. There is also strong evidence for antioxidant properties of resveratrol. Though we are unable to pinpoint mechanisms involved in this study, both anti-inflammatory and antioxidant effects (e.g., reduction in reactive oxygen species, activation of Sirtuin-1, etc. [48]) could play a role in alleviating symptoms of GWI.

Due to some evidence from animal research supporting a neuroprotective and anti-inflammatory role for luteolin [21], the compound has been proposed as a potential treatment for diseases involving inflammation [49,50]. However, very few studies have been conducted in human subjects. We found that luteolin did not appear to be effective in reducing GWI symptoms at either the lower (200 mg daily) or higher (400 mg daily) dosages. We have not found any published clinical trials examining luteolin as a possible treatment for GWI, FM, or ME/CFS.

Similar to luteolin, we found no evidence that fisetin (taken daily at 200 mg or 800 mg) reduces GWI symptoms. Though some findings from animal research suggest fisetin attenuates LPS/interferon-γ-induced neuroinflammation [51], human studies are very limited. We are not aware of any clinical trials with fisetin for treatment of GWI, FM, or ME/CFS.

### Limitations

In general, clinical trials involving botanical supplements must deal with challenges in the complexity and variability of compounds used [52]. Verification of content can be difficult as sourcing options and purity vary widely. Differences in sourcing, processing, storage, and multiple other factors may lead to disparities in botanical effects from study to study. Effects may also differ based on specific dosages tested in each study. We tested two dosages for each botanical in this study. It is unknown whether a lower, higher, or individually tailored dosage would be more effective in reducing GWI symptoms. In addition, dosage and duration of treatment were conflated in this study, given that participants on a higher-dose botanical had already been taking a lower dose of the same compound for the previous month.

Though our approach of testing multiple botanicals was advantageous in terms of efficiency, there were caveats to the design. Given the lengthy overall study commitment, sample sizes were small, which limits the generalizability of findings. Further, only men were recruited for this project, which prevents the generalizability of these results for women veterans with GWI. Some research suggests that GWI may be more common among women veterans of the Gulf War [53], and we recommend that future studies include both men and women if possible. Treatment periods were also relatively short, as participants received each dose of botanical for one month only in order to keep total initial participation duration within one year. It is possible that periods longer than one month are required to obtain full effect of the botanicals. Carryover effects are also possible with this design. Participants entered a placebo period immediately following a month of higher-dose botanical. Thus, after only two weeks of being off the higher dose of the previous botanical, symptom reports reflected the placebo condition of another botanical. The period of time between treatments may have been too short, and lingering effects of treatments or changes in symptom reporting over time may have occurred. Order effects are also a potential caveat with this design; however, the internal consistency for placebo severity scores across treatments (Cronbach’s alpha = 0.903) suggested there was no order effect on symptom reports.

No statistical analysis on the reported adverse events was conducted given the small incident rate. Though it appears the compounds overall are relatively well tolerated, larger sample sizes are required to draw more reliable conclusions regarding adverse effects. Finally, the single-item GWI severity VAS scales have not been validated as clinical trial outcomes. Common data elements for GWI were made available after the initiation of this study, and future studies should take advantage of those validated tools.

## 5. Conclusions

Rapid identification of treatments is crucial in order to improve the quality of life for individuals affected by GWI. The screening protocol employed in this study represents a method for efficiently assessing compounds already currently available (botanical, pharmaceutical, or otherwise) for potential efficacy in the treatment of GWI. To our knowledge, these studies are the first to be conducted with resveratrol, luteolin, and fisetin among veterans with GWI. Luteolin showed no effect on GWI symptoms, over and above the placebo response. Fisetin also showed no helpful effects at a lower or higher dosage. Resveratrol, however, was associated with lower GWI symptom severity at both the 200 mg/day and 600 mg/day dosages. While recognizing several limitations in this small study, we conclude that resveratrol should be prioritized in future GWI treatment efficacy studies.

## Figures and Tables

**Table 1:** Means (and standard deviations) and linear mixed models (LMM) results for the secondary outcomes of self-reported pain and fatigue. Results are presented separately for resveratrol (*n* = 10), luteolin (*n* = 11), and fisetin (*n* = 11).

	Baseline	Placebo	Lower Dose	Higher Dose	LMM
Resveratrol					
Pain	25.7 (20.8)	25.2 (20.5)	19.2 (15.6) *^#^	19.3 (14.7) *^#^	F (3, 556) = 7.2, *p* = 0.0001
Fatigue	29.5 (21.5)	29.15 (19.2)	27.1 (20.7) *	28.1 (21.0)	F (3, 659) = 8.7, *p* < 0.0001
Luteolin					
Pain	24.8 (17.2)	21.6 (14.0) *	23.3 (11.6)	20.8 (10.3) *	F (3, 690) = 5.9, *p* = 0.001
Fatigue	30.8 (22.5)	24.5 (20.0) *	27.9 (20.2) *	27.5 (21.5) *	F (3, 690) = 9.4, *p* < 0.0001
Fisetin					
Pain	35.0 (23.2)	33.2 (24.3)	31.2 (24.0)	31.1 (23.8)	F (3, 611) = 0.11, *p* = 0.955
Fatigue	43.9 (21.3)	42.8 (22.6)	41.8 (22.1)	43.3 (21.9)	F (3, 611) = 0.27, *p* = 0.845

* Significantly lower than baseline (*p* < 0.05); *^#^ significantly lower than baseline and placebo (*p* < 0.05).

**Table 2:** Incidence of self-reported adverse events.

	Resveratrol			Luteolin			Fisetin		
Adverse Event	P	LD	HD	P	LD	HD	P	LD	HD
Diarrhea	1	-	1	-	1	1	1	-	-
Dizziness	1	1	1	-	-	-	1	1	-
Upset stomach, lower GI	1	1	1	-	-	-	-	1	1
Worsening fatigue	-	-	-	-	-	-	1	1	2
Migraine	-	-	-	-	-	-	2	1	1
Headaches	-	1	-	-	-	-	1	1	1
Worsening GERD	-	-	-	1	1	1	-	-	1
Elevated creatinine kinase	-	-	-	-	-	-	-	1	1
Flushing	-	-	-	-	1	1	-	-	-
Elevated glucose	-	-	-	-	1	-	-	-	-
Worsening anxiety	-	-	-	1	-	-	-	-	-
Worsening headaches	-	-	-	-	-	-	-	-	1
Worsening nausea	-	-	-	-	-	-	-	-	1

P, Placebo; LD, lower dose; HD, higher dose; GERD, gastroesophageal reflux disease; GI, gastrointestinal.

## Data Availability

The datasets used and/or analyzed during the current study are available from the corresponding author upon reasonable request and the approval of the data owner.
